# Effects of Treatment with Citicoline Plus Nicotinamide Oral Solution on Retinal Ganglion Cells and Visual Pathways in Glaucomatous Impairment

**DOI:** 10.3390/ph19081179

**Published:** 2026-07-28

**Authors:** Vincenzo Parisi, Dario Romano, Amir Ali Aminoleslami, Lucia Ziccardi, Rebecca Prandini, Salvatore Martella, Luca Rossetti

**Affiliations:** 1 IRCCS—Fondazione Bietti, Via Livenza 6, 00198 Rome, Italylucia.ziccardi@fondazionebietti.it (L.Z.); 2 Departmental Faculty of Medicine, UniCamillus-Saint Camillus International University of Health Sciences, 00131 Rome, Italy; 3 Eye Clinic, ASST Santi Paolo e Carlo-San Paolo Hospital, University of Milan, 20142 Milan, Italy; 4 Department of Medicine and Health Sciences “V. Tiberio”, University of Molise, Via F. De Sanctis 1, 86100 Campobasso, Italy

**Keywords:** glaucoma, PERG, VEP, Citicoline, Nicotinamide

## Abstract

**Objectives**: To evaluate the retinal ganglion cell (RGC) function and neural conduction along the visual pathways after treatment with Citicoline plus Nicotinamide oral solution in patients with open angle glaucoma (OAG). **Methods:** Thirty OAG patients (mean age ± standard deviation: 62.588 ± 6.022 years, IOP < 18 mmHg with medical therapy) were enrolled. Of these, 20 OAG patients (providing 20 eyes) were treated with Citicoline plus Nicotinamide oral solution (Kron^®^, Omikron Italia) (Citicoline plus Nicotinamide group), and 10 OAG patients (providing 10 eyes) were treated with an oral placebo (Placebo group). In all participants, Humphrey 24-2 visual field (HFA 24-2), pattern electroretinogram (PERG), visual evoked potentials (VEP) and retinal nerve fiber layer thickness (RNFL-T) were assessed at baseline and at the end of the 3-month period of treatment. **Results:** PERG and VEP data obtained from 28 patients were included in the final analysis, while all the patients were included in the visual field and RNFL-T analyses. At baseline, both groups showed HFA 24-2 mean deviation (MD), PERG, VEP and RNFL-T values not statistically (ANOVA, *p* > 0.05) different. After 3 months of Citicoline plus Nicotinamide oral solution treatment, a significant (*p* < 0.05) increase in PERG P50-N95 and VEP N75-P100 amplitudes, and a significant (*p* < 0.05) shortening of VEP P100 implicit times were found. The shortening of VEP P100 implicit times was not significantly correlated (*p* > 0.01) with the increase in PERG P50-N95 amplitude. Conversely, in the Placebo group, no significant changes in PERG and VEP values were found. The HFA MD and RNFL-T changes were not significantly different (*p* > 0.05) between groups. **Conclusions:** In OAG patients, treatment with Citicoline plus Nicotinamide oral solution for three months induces RGC functional enhancement (increase in PERG amplitude) and improves the neural conduction along the visual pathways (shortening of VEP implicit time and increased amplitude), without significant changes in visual field or RNFL-T.

## 1. Introduction

The term glaucoma refers to a heterogeneous group of conditions characterized by death of retinal ganglion cells, structural defects to the optic nerve and visual field defects. Pathogenesis is multifactorial, and the first line therapy is based only on intraocular pressure (IOP) lowering strategies [1]. Early detection and correct treatment can slow down the progression of the disease, but retinal ganglion cell (RGC) death and damage to neural structures can still occur, due to deprivation of glucose, oxygen and other metabolic substances, neuroinflammation, oxidative stress and even autoimmune reactions [2,3,4].

In addition to IOP-lowering treatments, complementary strategies have been explored in the field of neuroprotection and neuroenhancement [5,6,7].

In particular, it is reported that, in glaucomatous patients, the treatment with Cytidine 5′-diphosphocholine (Citicoline), through a multifactorial mechanism of action (see as a review Oddone et al. [8] and Faiq et al. [9]), induces beneficial effects on visual field (VF) defects such as a reduction in the rate of progression [10,11], an enhancement of RGC function and neural conduction along the visual pathways [12,13], and an improvement in thalamo-cortical neural connections [14], with consequent better vision-related quality of life [15].

Another molecule attracting interest in the field of neuroprotection in glaucoma is Nicotinamide as the decline of Nicotinamide adenine dinucleotide (NAD+) in aged tissue has been associated with mitochondrial, neuronal and RGC dysfunction [16]. First on animals and then on humans, studies with Nicotinamide have gained relevance in the last few years [17]. In glaucoma patients, Nicotinamide treatment has shown to induce a short-term improvement in RGC function, evaluated by means of electroretinographic responses, and in VF after 12 weeks of treatment [18]. In addition, it is reported that glaucomatous eyes treated with Nicotinamide were more likely to experience improving test locations during VF examinations, compared with a placebo [19]. The Nicotinamide treatment mainly acts by enhancing mitochondrial functioning, with a more prominent effect on RGC activity [20].

Moreover, while Citicoline is generally well tolerable, even at high doses during a period of treatment of one year [14], the main issue in the studies with Nicotinamide treatment is related to the side effects (constipation, nausea, headaches and tinnitus) observed at the high dosages used (1.5 to 3.0 g/day) in up to one third of the participants [19].

To achieve different effects and take advantage of the complementary and consequently synergistic actions of the two molecules such as the structural and functional action of Citicoline [8,9] and the RGC mitochondrial activity of Nicotinamide [20], a new patented oral formula (oral solution with high bioavailability) combining Citicoline and Nicotinamide (Kron^®^, Omikron Italia s.r.l.) is commercially available. Given the multifactorial pathogenesis of glaucoma, combination therapy targeting different neurodegenerative pathways may increase the likelihood of achieving meaningful neuroprotection. Therefore, combination therapies acting on complementary pathways may provide greater neuroprotective benefits than individual agents alone, particularly when initiated during the early stages of the disease [7]. Beyond the potential biological synergy, combining the two molecules into a single oral formulation may theoretically enhance patient adherence, which is well known to be reduced in complex treatment regimens [21,22].

Therefore, the aim of this study was to assess whether in OAG patients, a 3-month treatment with Citicoline plus Nicotinamide in oral solution at a dosage of 400 mg/day and 150 mg/day, respectively, could improve RGC function (assessed by pattern electroretinogram (PERG) recordings) and the neural conduction along both large and small axons forming the visual pathways (assessed by visual evoked potentials (VEP) recordings).

In addition, we aimed to establish whether the possible improvement in the neural conduction along the visual pathways was dependent on changes in RGC function and whether concomitant changes in VF defects or of retinal nerve fiber layer thickness (RNFL-T) are also detectable.

## 2. Results

At follow-up, V.P. analyzed the PERG and VEP bioelectrical responses of all enrolled patients detected at baseline and without knowing whether the tested patient had taken the placebo or Citicoline plus Nicotinamide.

Based on the above-mentioned criteria of PERG acceptance (SNR > 2), at baseline, since PERG SNR < 2 was detected in one enrolled patient and this PERG response was considered “not acceptable”, the data of this patient were excluded from the analysis. For the same reason, at the follow-up, the PERG response obtained in one enrolled patient was considered “not acceptable” and therefore the data of this patient were excluded from the analysis. Considering the secondary aim of our work (“In addition, we aimed to establish whether the possible improvement in the neural conduction along the visual pathways was dependent or not on the changes in RGC function”), for those patients whose PERG responses were excluded, their VEP responses were not included in the analysis.

When the key was opened, we found that the patients excluded from the analysis of PERG and VEP data had been assigned to the group of patients treated with Citicoline and Nicotinamide.

Therefore, considering the two patients excluded for not acceptable PERG responses, from the twenty patients initially assigned to the Citicoline and Nicotinamide group, the electrophysiological data obtained in 18 patients were considered in the analysis of PERG and VEP responses detected at baseline and at follow-up. A CONSORT diagram reporting details concerning screening, enrolment, randomization and data analysis is available as Supplementary Figure S1 [23]. Baseline characteristics and glaucoma medications of the study groups are available as Supplementary Table S1.

Thus, regarding the PERG and VEP findings, the Citicoline plus Nicotinamide group consisted of 18 OAG patients (mean ± SD age 63.692 ± 6.713 years, mean ± SD IOP 13.5 ± 1.8 mmHg), providing 18 eyes.

Since none of patients from the Placebo group was excluded, this group consisted of 10 OAG patients (mean ± SD age 62.200 ± 6.363 years, mean ± SD IOP 13.0 ± 2.1 mmHg), providing 10 eyes.

In both groups, the mean IOP did not show statistically significant (*p* > 0.05) changes throughout the entire follow-up of the study.

Both the Citicoline plus Nicotinamide oral solution and the placebo were well tolerated with no reported adverse events throughout the study.

**Figure 1:**
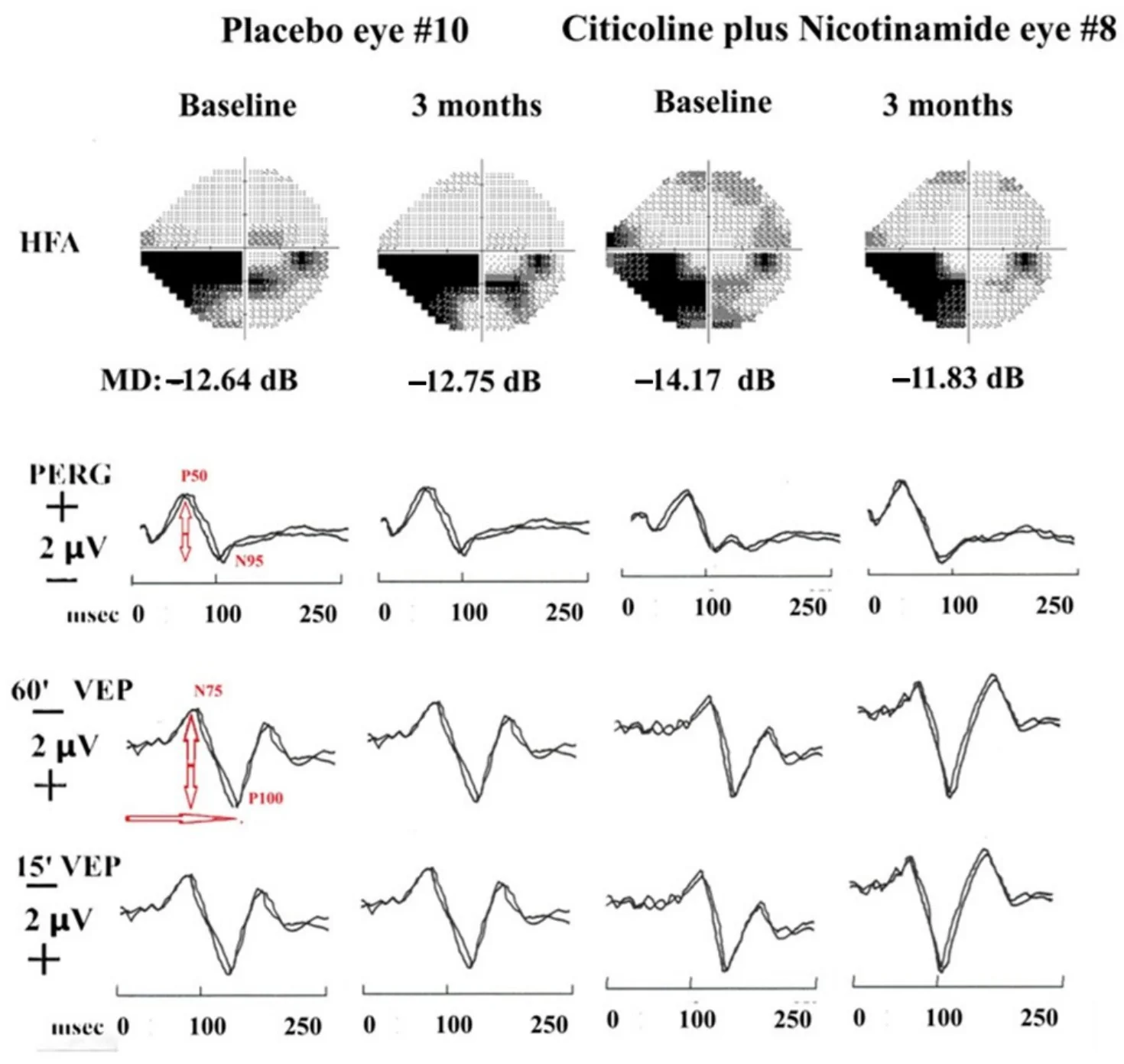
Examples of Humphrey Field Analyzer 24-2 (HFA 24-2) at baseline and after 3 months of treatment with Placebo (Placebo eye #10) or Citicoline plus Nicotine oral solution (Citicoline plus Nicotinamide eye #8). It should be noted that in the Citicoline plus Nicotinamide eye, at the end of treatment, an improvement in mean deviation (MD) of 2.34 dB was detected, whereas in the Placebo group eye, at the end of treatment, a decrease in MD of −0.11 dB was observed. Examples of PERG and VEP recordings at baseline and after 3 months of treatment performed in Placebo eye #10 and in Citicoline plus Nicotinamide eye #8. In comparison to baseline condition, after treatment with Citicoline plus Nicotinamide, an increase in PERG P50-N95 amplitude (↕), a shortening of 60′ and 15′ VEP P100 implicit times (→), and an increase of 60′ and 15′ VEP amplitudes (↕) were found. After treatment with the placebo, PERG P50-N95 amplitudes and VEP P100 implicit times and N75-P100 amplitudes were similar to baseline values. 60′ and 15′ refer to visual stimuli in which each check subtended 60 min and 15 min of the visual arc, respectively.

Figure 1 shows examples of HFA 24-2, PERG and VEP recordings performed in one OAG patient treated with Citicoline plus Nicotinamide oral solution for 3 months and in one OAG patient that was treated with the placebo for 3 months.

### 2.1. PERG Results

As shown in Table 1, at baseline condition, the Citicoline plus Nicotinamide and Placebo groups showed PERG A values that were not significantly (*p* > 0.05) different.

**Figure 2:**
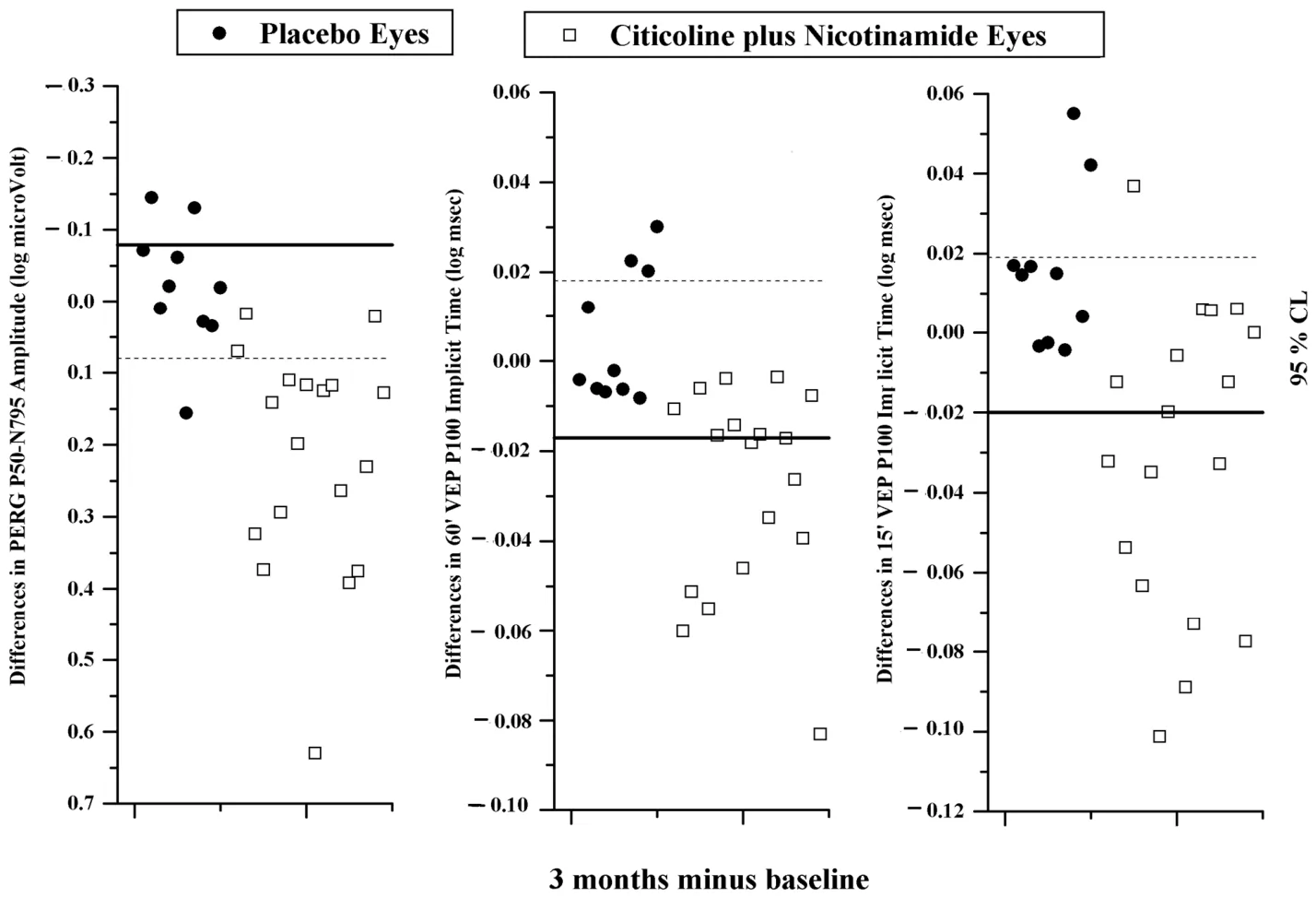
Plot of individual changes (3 months minus baseline) observed in OAG patients treated with Citicoline plus Nicotine oral solution (Citicoline plus Nicotinamide, 13 eyes), or the placebo (Placebo group, 10 eyes). The percentage of unmodified eyes (within the 95% confidence test–retest limit, CL), eyes with improvement (values over the 95% CL—dashed line—for PERG P50-N95 amplitudes and values under the 95% CL—solid line—for VEP P100 implicit times) and eyes with worsening (values under the 95% CL—solid line—for PERG P50-N95 amplitudes and values over the 95% CL—dashed line—for VEP P100 implicit times) is reported in Table 2. 60′ and 15′ refer to visual stimuli in which each check subtended 60 min and 15 min of the visual arc, respectively.

Regarding the individual differences (3 months minus baseline), the majority (15/18, 83.33%) of eyes treated with Citicoline plus Nicotinamide showed an improvement in P50-N95 As; in contrast, the majority (7/10, 70.00%) of eyes treated with the placebo showed unchanged values (see Table 2 and Figure 2).

Table 3 reports the mean values of individual differences detected at 3 months minus baseline. Significant (*p* < 0.01) differences between the Citicoline plus Nicotinamide and Placebo groups were found.

On average, as presented in Table 1, in the Citicoline plus Nicotinamide group, a significant (*p* = 0.005) increase in PERG A values was detected at 3 months with respect to the baseline values. In the Placebo group, at the follow-up (3 months), non-significant (*p* > 0.05) differences with respect to baseline values were found.

### 2.2. VEP Results

At baseline condition, the Citicoline plus Nicotinamide and Placebo groups, showed similar values (*p* < 0.05) of 60′ and 15′ VEP ITs and As.

As reported in Table 2 and in Figure 2, considering the individual differences (3 months minus baseline), the majority (ranging from 55.56% for 15′ VEP ITs to 66.67% for 15′ and 60′ VEP As) of eyes treated with Citicoline plus Nicotinamide showed an improvement of 60′ and 15′ VEP ITs and As; in the majority (70–80%) of eyes treated with the placebo, unchanged values of 60′ and 15′ VEP ITs and As were detected.

Table 3 reports the mean values of individual changes (3 months minus baseline), and significant (*p* < 0.01) differences between the Citicoline plus Nicotinamide and Placebo groups were found.

In the Citicoline plus Nicotinamide group, the individual differences of 60′ and 15′ VEP ITs were non statistically (60′: r = 0.157, *p* = 0.487; 15′: r = 0.010, *p* = 0.968) linearly correlated with the corresponding individual differences in PERG As.

On average, after 3 months of treatment with respect to baseline, in the Citicoline plus Nicotinamide group, significant shortening of 60′ and 15′ (*p* = 0.027 and *p* = 0.014, respectively) VEP ITs values and significant increases of 60′ and 15′ (*p* = 0.037 and *p* = 0.043, respectively) VEP As values were observed. In the Placebo group, at the follow-up (3 months) non-significant (*p* > 0.05) differences with respect to baseline were found (see Table 1).

### 2.3. Visual Field Results

The HFA data obtained at baseline and at 3-month follow-up are available for the 20 and 10 OAG patients initially assigned to the Citicoline plus Nicotinamide and Placebo groups, respectively.

At baseline, the mean HFA 24-2 MD values detected in the 20 patients initially assigned to Citicoline plus Nicotinamide group (−10.208 ± 3.671 dB) were not significantly [f(1, 29) = 0.25, *p* = 0.623] different with respect to those of initially assigned to the Placebo group (−9.510 ± 3.516 dB) (−9.510 ± 3.516 dB).

The MD mean differences (3 months minus baseline) observed in the Citicoline plus Nicotinamide group were not significantly different (*p* > 0.05) from those found in the Placebo group.

The probability of improvement beyond the upper 95% test–retest limits was 14.2% (estimate [95% confidence intervals] in the Placebo group and 15.4% in the treated group; odds-ratio 1.10 [0.47, 2.55], *p* = 0.8267108).

For MD, the average change between baseline and 3 months after the initiation of treatment was −0.26 [−1.07, 0.54] dB (*p* = 0.525) for the Placebo group and 0.00 [−0.52, 0.53] dB (*p* = 0.9895682) for the Citicoline plus Nicotinamide group. The difference in change from baseline between the two groups was 0.27 [−0.69, 1.23] dB (*p* = 0.589).

For TD, the average change between baseline and 3 months after the initiation of treatment was −0.18 [−1.08, 0.72] dB (*p* = 0.6960) for the Placebo group and 0.00 [−0.59, 0.59] dB (*p* = 0.9879324) for the Citicoline plus Nicotinamide group. The difference in change from baseline between the two groups was 0.18 [−0.89, 1.26] dB (*p* = 0.7403).

**Figure 3:**
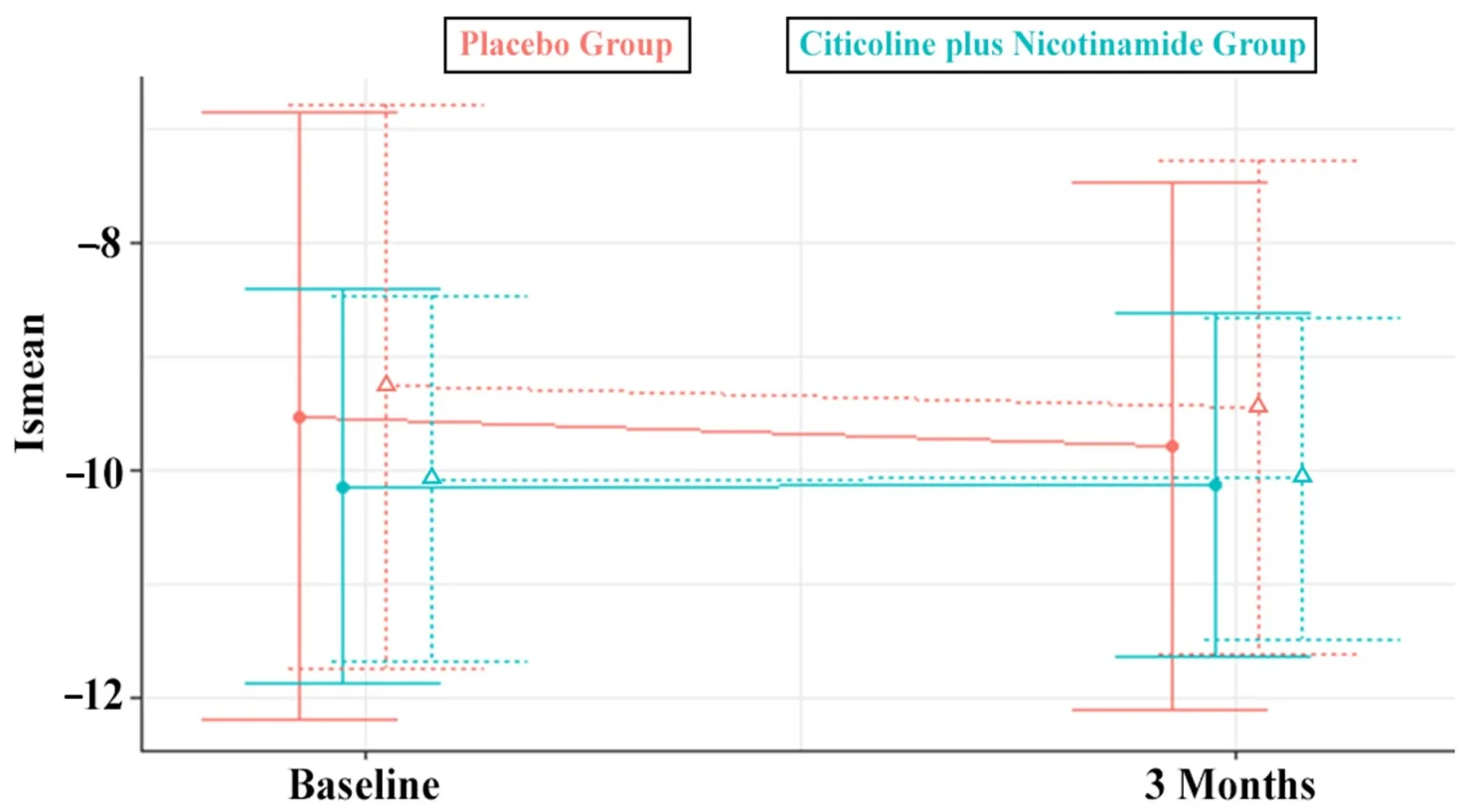
Estimates (dots and lines) and 95% confidence intervals (vertical bars) for the mean deviation (● and solid lines) and total deviation (∆ and dotted lines).

The estimates from MD and TD are reported in Figure 3.

### 2.4. RNFL-T Results

The RNFL-T data obtained at baseline and at 3-month follow-up are available for the 20 and 10 OAG patients initially assigned to the Citicoline plus Nicotinamide and Placebo groups, respectively.

At baseline, the mean RNFL-T values detected in the 20 patients initially assigned to the Citicoline plus Nicotinamide group (59.150 ± 9.224 micron) were non significantly [f(1, 29) = 0.68, *p* = 0.416] different with respect to those of initially assigned to the Placebo group (55.600 ± 14.269 micron).

At the end of the study, the RNFL-T mean changes observed in the Citicoline plus Nicotinamide group were not significantly different (*p* > 0.05) when compared to the Placebo group.

## 3. Discussion

Our aim was to assess whether in OAG patients the treatment (3 months) with Citicoline plus Nicotinamide in oral solution could improve the RGC function (assessed by PERG amplitude changes) and the neural conduction along both large and small axons forming the visual pathways (assessed by 60′ and 15′ VEP changes) with associated changes in visual field defects or of RNFL-T.

Our results suggest that in OAG patients the treatment for 3 months with Citicoline (dosage of 400 mg/day) plus Nicotinamide (dosage of 150 mg/day) oral solution induced an enhancement of RGC function (PERG improvement), likely due to the mechanism of action of both components. By the same treatment, better neural conduction along the visual pathways (VEP improvement), likely due to the neuromodulator effect of Citicoline, is also evident. This treatment, when performed for 3 months, does not induce significant changes in visual field defects or in RNFL-T. However, in the present study, when both eyes met the inclusion criteria, the eye with the greater baseline RGC dysfunction was selected for analysis. Since we previously reported that treatment with Citicoline eye drops produced the greatest PERG improvement in eyes with more severe baseline RGC dysfunction [13], this selection criterion may have influenced the magnitude of the post-treatment functional improvement observed in our cohort.

In recent years, increasing attention has been devoted to evaluating the effects of combining different neuroprotective agents to target the multiple pathogenic mechanisms involved in glaucomatous neurodegeneration. In experimental models of glaucoma, for example, choline combined with vitamins B6, B9, and B12 has been shown to reduce RGC loss, supporting the rationale for multimodal neuroprotective strategies [24]. Furthermore, Citicoline combined with Nicotinamide in aqueous solution has shown greater efficacy in restoring RGC function in a murine model compared to the individual molecules alone, suggesting a potential synergistic effect of the combination [25]. This enhanced effect is biologically plausible given their complementary mechanisms of action: Citicoline exerts neuroprotective effects at the level of RGCs, the optic nerve head, and the post-chiasmatic visual pathways, whereas Nicotinamide primarily improves mitochondrial function and cellular bioenergetics. Although the present study was not designed to formally assess synergism, the observed clinical benefits are consistent with this preclinical evidence and support the hypothesis that the combined oral formulation of Citicoline and Nicotinamide may provide greater neuroprotective effects than either molecule alone.

We observed that no patients treated with Citicoline plus Nicotinamide oral solution reported any adverse events throughout the study. Therefore, considering the lack of adverse effects reported in previous studies performed exclusively with Citicoline oral solution [10,14], the administration of Nicotinamide (at the dosage of 15 mg/mL) can be considered safe and well tolerated. This result strengthens the recent evidence, that associate Nicotinamide exposure with a reduced risk of developing OAG, with a supplementation dosage [26], even though a direct comparison with other trials using higher concentrations is not feasible.

### 3.1. Effect of Citicoline Plus Nicotinamide Oral Solution on RGC Function (PERG Data)

In the present study, we evaluated the RGC function by PERG recordings by using visual stimuli (48′ of visual arc) according to the ISCEV standards [27]. PERG has been shown to be an optimal tool for the detection of RGC dysfunction in glaucoma, being able to identify it before manifesting glaucomatous visual field damage [28,29].

In our OAG patients, after treatment with Citicoline plus Nicotinamide oral solution, the RGC function was enhanced, as indicated by increases in PERG As.

This agrees with the PERG results reported in our previous study performed by using Citicoline oral solution [14].

Also, by administrating Nicotinamide at the dosage of 1.5–3.0 g/day, an increase in amplitude of the Photopic Negative Response (PhNR) (another electrophysiological method to assess the RGC function [30]) was observed [18], and this suggests that Nicotinamide can induce a functional enhancement of glaucomatous RGCs.

Indeed, this study has been carried out by administering an optimized dosage of both the molecules (Citicoline 400 mg/day and of Nicotinamide 150 mg/day), differing from that used in other studies [10,14,18], and it was particularly interesting that increases in PERG As were equally observed.

These results can be explained through the complementary and consequently synergistic effect of the two molecules when administered in a unique combination in oral solution with high bioavailability (Kron^®^, Omikron, Milan, Italia).

In summary, Citicoline exerts a multifactorial mechanism of action: it functions as an endogenous precursor in the biosynthesis of phosphatidylcholine, thereby contributing to the preservation of phospholipid membrane integrity and cellular redox balance. It also supports mitochondrial homeostasis, and by improving cardiolipin, exerts modulatory effects on both cholinergic and dopaminergic neurotransmission. By promoting the synthesis of key membrane components such as phosphatidylcholine and sphingomyelin, Citicoline helps with maintaining neuronal membrane stability. Its ability to enhance sphingomyelin production, to stabilize RGC axonal membranes, and to limit the release of free fatty acids contributes to the attenuation of oxidative stress and neuroinflammatory responses. In parallel, Citicoline has been shown to improve mitochondrial function [8,9].

The effects of Citicoline on RGC function are well known, both in glaucoma and in non-arteritic ischemic optic neuropathy [8].

Regarding its direct effects on RGCs, experimental studies (using retinal culture models) demonstrated that Citicoline can reduce RGC degeneration [31,32]. Moreover, Matteucci et al. reported that, in retinal cultures exposed to glutamate or high-glucose conditions (established models of neurodegeneration) Citicoline administration decreases pro-apoptotic signaling associated with synaptic damage [33].

Nicotinamide has a protective effect on mitochondrial activity [17]. Considering the effects of Nicotinamide on RGCs, there are several experimental studies supporting that this molecule could prevent the RGC mitochondrial damage or dysfunction.

Williams et al. [17] used RNA sequencing of RGCs from DBA/2J mice of different ages, identifying mitochondrial dysfunction as an early event in glaucoma. Subsequent studies showed that an age-related decline in Nicotinamide Adenine Dinucleotide (NAD, a key molecule in energy and redox metabolism) contributes to RGC vulnerability. In the same animal model (DBA/2J mice), NAD restoration, through Nicotinamide treatment, induced significant neuroprotection, highlighted by a reduction in synaptic loss, of RGC degeneration and optic nerve damage, and this was associated with an improvement in retinal function. Similar protective effects were observed with genetic or gene therapy approaches enhancing NAD levels, preserving dendritic structure and synaptic integrity [17,20,25,34].

The first evidence of synergy in a glaucoma mouse model has shown that the combination based on Citicoline and Nicotinamide, administered in aqueous solution, is able to exert a protective effect on mitochondrial function and consequently on the density of RGCs [34].

### 3.2. Effect of Citicoline Plus Nicotinamide Oral Solution on Neural Conduction Along the Visual Pathways (VEP Data)

In this study, we assessed the neural conduction along the visual pathways by pattern VEP recordings in response to checks subtending 60′ and 15′ of visual arc, following the ISCEV standards [35].

In glaucomatous patients, the visual stimulation with checks subtending 60′ and 15′ of visual arc allows us to assess the neural conduction along the large (60′) and small (15′) axons forming the visual pathways [34], and it is worth noting that when VEP are recorded in response to the 15′ pattern stimuli, a sensitivity and specificity of 100% and a significant correlation with visual field HFA MD has been found in OAG patients [36].

After treatment with Citicoline plus Nicotinamide oral solution, a significant shortening of 60′ and 15′ VEP ITs and a significant reduction in 60′ and 15′ VEP As was found, suggesting that this treatment can improve the neural conduction along both large and small axons forming the visual pathways.

Although in this study we performed a 3-month treatment with Citicoline at the dosage of 40 mg/mL, the observed VEP changes agree with those reported in our previous study performed using a higher dosage (50 mg/mL) of Citicoline in oral solution for a longer period of follow-up (12 months) [14].

It is worth noting that when glaucomatous patients are treated exclusively with Nicotinamide (500 mg daily for six months) using the same method performed in this study (visual stimulation with checks subtending 60′ and 15′ of visual arc), the VEP responses detected after the treatment were not significantly modified with respect to pre-treatment ones [18].

Thus, based on previous evidence [34] suggesting that the exclusive treatment with Nicotinamide does not induce an improvement of neural conduction along the visual pathways, we could deduce that the observed VEP changes detected after Citicoline plus Nicotinamide treatment can be ascribed exclusively to the effect of Citicoline, as previously described [14].

To explain the effects of Citicoline on the glaucomatous neural conduction along large (detected by 60′ VEP) and small (detected by 15′ VEP) axons forming the visual pathways, we must take into consideration that, as previously reported [36], the glaucomatous visual field defect can be ascribed to two sources of impairment: one at the RGC level, and one at post-retinal visual pathways level. The latter can be due to several factors such as synaptic impairment between RGC axons and the neural elements of Lateral Geniculate Nucleus (LGN) with consequent neural degeneration of both magnocellular and parvocellular LGN layers [37,38], abnormal thalamo-cortical neural connectivity [39], cortical atrophy [40], and a demyelinating process within optic radiations [41]. This impairment occurring in the visual pathways can be detectable by the increase in VEP ITs and the increase in retino-cortical time (RCT, an electrophysiological index of the post-retinal neural conduction along the visual pathways) observed in OAG patients [35].

Thus, the VEP changes after treatment with Citicoline plus Nicotinamide, which are independent of the increase in RGC function (as deducible from the lack of significant correlations between the PERG As increase and IT 60′ and 15′ VEP ITs shortening) can be explained based on a particular property of Citicoline: a neuromodulator effect due to dopaminergic-like activity [9].

In fact, based on this property, Citicoline administrated in oral solution could provide an exogenous supplementation of neurotransmitters to act on the synaptic impairment between RGC axons and LGN, with consequent potentiation of these synapses. This induces an improvement of thalamo-cortical connectivity, as documented by the increase in the density of optic radiation fibers detected by Brain Diffusion Tensor Imaging (DTI)-Magnetic Resonance Imaging (MRI) performed in glaucomatous patients before and after 12 months of treatment with Citicoline oral solution [14].

Nevertheless, considering that in experimental models the Citicoline treatment induces axonal remyelination [42,43], and that demyelinating process in human post-mortem glaucomatous optic nerve has been observed [44], we cannot entirely exclude that Citicoline oral solution could promote an axonal remyelination process with consequent improvements in neural conduction along the visual pathways, as suggested by VEP improvement.

### 3.3. Effect of Citicoline Plus Nicotinamide Oral Solution on HFA-MD and RNFL-T

The two studied groups showed non-significant differences in the mean values of the differences (3 months minus baseline) of HFA and RNFL-T. This is not in agreement with previous results in which the mean MD differences observed in OAG patients treated with Citicoline in oral solution were significantly greater than those detected in OAG patients treated with the placebo [10,11].

Nevertheless, these changes are observed after one year of treatment, and therefore, we cannot exclude that with a longer follow-up period than that of this study (3 months), even using Citicoline and Nicotinamide in oral solution, significant visual field changes should be identifiable. Even though, consistent with the published literature on Nicotinamide, we were able to find an improvement in inner retinal function with electroretinography, our data did not show significant changes in VF examinations. Notably, while De Moraes and colleagues [19] found a significantly higher probability of improving test locations with Nicotinamide compared with the placebo, in our population study, treated patients had a non-significant higher chance of sensitivity improvements. It is noteworthy that in this work, a higher dose of Nicotinamide (up to 3000 mg) added to an equivalent dose of pyruvate was tested, which led to gastrointestinal side effects in one-third of treated patients, while our lower dosage oral solution was well tolerated by each participant. No significant changes in OCT results were observed in either the Citicoline plus Nicotinamide group or the Placebo group, even though structural data from posterior pole scans, such as ganglion cell layer thickness, were not extracted. Given the relatively short follow-up period, structural parameters were included primarily to exclude structural changes that could confound the interpretation of functional outcomes, rather than to assess the neuroprotective effects of the investigated treatment.

## 4. Materials and Methods

### 4.1. Study Design and Participants

This was a randomized, prospective, double-blind study. The protocol adhered to the Declaration of Helsinki. It was approved by the Local Ethic Committee on 13 September 2024 (Comitato Etico Milano Area 1, CET 212-2024) and registered at ClinicalTrials.gov (NCT06333236).

All patients were informed about the study outcomes and gave written informed consent prior to participation.

A total of thirty patients with a diagnosis of OAG and risk of glaucoma progression were selected from a cohort of 130 individuals, based on specific inclusion criteria.

The inclusion criteria were as follows: VF deficit, assessed using Humphrey Field Analyzer (HFA, Carl Zeiss Meditec, Inc., Dublin, CA, USA) 24-2 program, with a mean deviation (MD) ranging from −6 to −16 dB. To be considered at risk of progression patients must have at least two of the following risk factors: pseudoexfoliation syndrome, history of disk hemorrhage, thinner cornea, myopia (<−6 diopters), family history of advanced glaucoma, IOP > 21 mmHg at baseline, and MD at 24-2 VF examination < −6 dB at baseline [45,46].

Test reliability was ensured by including only examinations with fixation losses, false positives, and false negatives, each below 20% [47]; best-corrected visual acuity (BCVA) between 0.0 and 0.1 logMAR; characteristic signs of glaucomatous optic nerve damage as documented by color stereo-photographs; and corresponding visual field defects. Optic disc photos and visual fields were independently reviewed by two expert glaucoma specialists (D.R. and A.A.A.). In case of disagreement, a third glaucoma specialist (L.R.) adjudicated, and the final classification was assigned by majority vote. Further inclusion criteria were as follows: refractive error between −3.00 and +3.00 spherical equivalent; absence of any history or documented evidences of diseases affecting the cornea, lens, macula, or retina as well as absence of diabetes, optic neuritis, or any neurological conditions involving the visual pathways; pupil diameter greater than 3 mm without the use of mydriatics; central corneal thickness within 500–600 µm, as measured using a specular microscope (Konan CellChek SL, Konan Medical Inc., Hyogo, Japan); and unchanged IOP-lowering eye drops (with IOP < 18 mmHg) for at least eight months prior to baseline evaluation—and throughout the study—since PERG responses can be affected by pharmacological IOP reduction [48,49].

Based on the above-mentioned inclusion criteria, 30 patients were selected and randomized (see below).

We considered 30 OAG eyes from 30 patients; when both eyes met inclusion criteria, only one eye per patient was included in the study. The selected eye was the one exhibiting greater RGC dysfunction, as indicated by lower PERG P50-N95 amplitude values (see below).

At baseline, the 30 selected patients were randomly assigned to two age-similar groups, with 20 individuals assigned to the Citicoline plus Nicotinamide group and 10 to the Placebo group.

Randomization, performed using an electronic system (https://www.randomizer.org; accessed on 2 February 2025), was stratified according to age, sex, IOP, HFA MD, and VEP P100 implicit time. Allocation remained masked to all investigators until the completion of the follow-up period and after the analysis of the PERG and VEP bioelectrical responses. S.M. was the only one aware of the treatment allocations, as he was responsible for dispensing the masked study bottles to participants. To preserve allocation concealment, he was not involved in any aspect of data collection, clinical assessment, or data analysis. After this process, the key was opened and it was identified whether each patient was assigned to the Placebo or Citicoline plus Nicotinamide group.

From baseline to month 3, in addition to IOP lowering drops, each Citicoline plus Nicotinamide group patient received 10 mL/day of Kron^®^, Omikron Italia (oral solution containing Citicoline free acid 40 mg/mL; Nicotinamide 15 mg/mL; water; fructose; sodium lactate; citric acid; potassium sorbate). Similarly, each Placebo group patient received 10 mL/day of the vehicle solution, with the same packaging, smell and taste, but without Citicoline and Nicotinamide.

Oral solution had to be taken during the morning, and the adherence to treatment was assessed via questionnaires distributed at each visit asking patients to self-grade their adherence as good or poor if they missed less or more than 30% of doses, respectively. Moreover, at the end of the study, participants were required to return the used bottles, allowing the study staff to verify the reported treatment compliance. Poor adherence was considered a criterion for excluding the patients from the study.

### 4.2. Electrophysiological Assessment

In all enrolled eyes, electrophysiological examination was performed at baseline and at the 3-month follow-up by R.P. In agreement with previously published studies [14,35], PERG and VEP recordings were performed using the methods described below.

Subjects were seated in a semi-dark, acoustically isolated room in front of the display surrounded by a uniform field of luminance of 5 cd/m^2^. Prior to the experiment, each subject was adapted to the ambient room light for 10 min, with a pupil diameter, measured by a ruler, of approximately 5 mm. Mydriatic or miotic drugs were never used. Stimulation was monocular after occlusion of the fellow eye. PERG and VEP recordings were performed with full correction of refraction at the viewing distance. A small red fixation target, subtending a visual angle of approximately 0.5° (estimated after considering spectacle-corrected individual refractive errors) was placed at the center of the pattern stimulus. At every PERG and VEP examination, each patient positively reported that he/she could clearly perceive the fixation target.

#### 4.2.1. PERG Recordings

According to the ISCEV standards [27], visual stimuli were checkerboard patterns (contrast 80%, mean luminance 110 cd/m^2^) generated on a television monitor and reversed in contrast at the rate of two reversals per second. At a viewing distance of 114 cm, the check edges subtended 48 min (48′) of visual angle, while the monitor screen subtended 18°.

PERG signals were recorded via Ag/AgCl skin electrodes placed on the lower eyelid. The ground electrode was in Fpz [50]. A bipolar configuration was used, with the active electrode on the stimulated eye and the reference electrode on the patched eye. Interelectrode resistance was lower than 3000 Ω. The signal was amplified (gain 50,000), filtered (band pass 1–30 Hz), and averaged with an automatic rejection of artifacts (200 events free from artifacts were averaged for every trial) by using the Retimax Advanced Plus system (CSO, Florence, Italy). Analysis time was 250 ms.

In the transient PERG response, the P50 and N95 components were identified, and peak-to-peak P50–N95 amplitudes (PERG A) were measured.

#### 4.2.2. VEP Recordings

According to the ISCEV standards [51], visual stimuli were checkerboard patterns (contrast 80%, mean luminance 110 cd/m^2^) generated on a television monitor and reversed in contrast at the rate of two reversals per second. At a viewing distance of 114 cm, the check edges subtended 60 min (60′) and 15 min (15′) of visual angle, while the monitor screen subtended 18°.

Cup shaped electrodes of Ag/AgCl were fixed with collodion in the following positions: active electrode in Oz [50], and reference electrode in Fpz. The ground was on the left arm. Interelectrode resistance was kept below 3000 Ω. The bioelectric signal was amplified (gain 20,000), filtered (band pass 1–100 Hz) and averaged with an automatic rejection of artifacts (200 events free from artifacts were averaged for every trial) using the Retimax Advanced Plus system (CSO, Florence, Italy). Analysis time was 250 ms.

The transient VEP response is characterized by a number of waves with three subsequent peaks of negative, positive, and negative polarity, respectively. In visually normal subjects, these peaks have the following implicit times: 75, 100, and 145 ms (N75, P100, N145). In the transient VEP response, the N75 and P100 components were identified, and P100 implicit time (VEP IT) and peak-to-peak N75–P100 amplitudes (VEP A) were measured.

Each recording session included a minimum of two and a maximum of six repeated acquisitions to verify waveform reproducibility. Based on previous findings [34], intra-individual variability was estimated as ±2 ms for VEP P100 implicit time and ±0.18 µV for PERG amplitude. Two recordings were considered reproducible if the difference fell within these ranges. When necessary, additional recordings were performed, never exceeding six per session. In addition, the two reproducible recordings provided the data for the test–retest intra- and inter-individual variability of the entire cohort of enrolled patients.

At the follow-up, PERG and VEP bioelectrical responses were analyzed by an expert in electrophysiological examination of the visual system (V.P.), with particular attention to the PERG responses in relationship to the signal-to-noise ratio (SNR).

In each subject or patient, the signal-to-noise ratio (SNR) of PERG responses was assessed following a previously published method [36] by measuring a ‘noise’ response while the subject fixated at an unmodulated field of the same mean luminance as the stimulus. At least two “noise” records of 200 events each were obtained, and the resulting grand average was considered for measurement. The peak-to-peak amplitude of this final waveform (i.e., average of at least two replications) was measured in a temporal window corresponding to that at which the response component of interest (PERG P50-N95) was expected to peak. The SNR for this component was determined by dividing the peak amplitude of the component by the noise in the corresponding temporal window. In all subjects and patients, we accepted PERG responses with SNR > 2.

For statistical analysis, the averaged values of PERG and VEP parameters from reproducible traces were considered.

### 4.3. Visual Field (VF) Examination

Visual field examination was performed with a Humphrey Field Analyzer (HFA), using the 24-2 SITA Standard pattern. During the baseline visit and during the final visit, each patient underwent 4 visual field tests. Visual field examinations were clustered to improve the ability to detect progression or improvement [52,53]. All visual field examinations had to be reliable, with false positive < 15% and fixation losses < 20%. If one of the clustered examinations did not meet the reliability criteria, it was repeated once. In cases of persistent unreliability, the patient was excluded from the study. VF global indices [mean deviation (MD), pattern standard deviation (PSD) visual field index (VFI) and total deviation (TD)] and pointwise sensitivities were extrapolated from each exam.

### 4.4. Retinal Nerve Fiber Layer Thickness (RNFL-T) Assessment

Peripapillary RNFL was assessed by using a Spectral-domain Optical Coherence Tomography (Sd-OCT) device [Heidelberg Spectralis (version 1.12.1.0, Heidelberg Engineering, Heidelberg, Germany] according to the APOSTEL recommendations [54]. Circular scans centered on the optic nerve head (ONH) were acquired using the peripapillary RNFL protocol. The characteristics of Sd-OCT evaluation are reported extensively in our previous work [32]. In the Sd-OCT results, RNFL-T was measured by using a circular scan with a diameter of 3.5 mm around the optic nerve. All OCT examinations were required to be of high quality, with a Q-score > 20 and no truncated or inverted scans.

### 4.5. Statistical Analysis

The primary endpoint was the change in 15′ VEP P100 implicit time in the Citicoline plus Nicotinamide group relative to the Placebo group. Sample size estimation was based on previous data [14], which reported a 15′ VEP P100 implicit time of 132.127 ± 8.571 ms at baseline and of 121.9070 ± 7.111 ms after 6 months of treatment with Citicoline oral solution. Considering the before–after study design and assuming an alpha error of 5% and a power of 80%, 6 subjects per group were required. Considering a dropout rate of 20%, the final target was adjusted to at least 8 participants per group.

Test–retest data (obtained in the group of OAG patients evaluated in this study) of PERG and VEP results were expressed as the mean difference between two recordings obtained in separate sessions ± SD of this difference. A 95% confidence limit (CL, mean ± 2 SD) of test–retest variability in OAG patients was established assuming a normal distribution.

At baseline, the IOP, HFA 24-2, PERG and VEP parameters values observed in the Citicoline plus Nicotinamide and Placebo groups were compared by one-way analysis of variance (ANOVA) for repeated measures. In each group, IOP, PERG As, VEP ITs and As values observed at the end of treatment were compared to baseline ones by one-way analysis of variance (ANOVA) for repeated measures. After treatments, the differences observed in individual OAG eyes with respect to the baseline values, were calculated by performing a logarithmic transformation to better approximate a normal distribution. The differences in PERG, VEP, and RNFL-T values observed between the Citicoline plus Nicotinamide and Placebo groups were evaluated by ANOVA. A *p*-value less than 0.05 was considered statistically significant.

For VF evaluation, the average change between baseline and post-treatment follow-up was tested with a linear mixed effect model, with random intercepts and slopes. An interaction term modeled the difference in post-treatment improvement between eyes on placebo and treatment. The same model was used for mean deviation (MD) and total deviation (TD) point-wise values.

To detect the probability of improvement, the baseline test data (4 per patient) were used to generate personalized upper 95% test–retest limits. These limits were used to determine improvement beyond chance for different baseline sensitivity levels. For each test location in each eye, we calculated the average baseline sensitivity as the arithmetic mean of the four baseline sensitivity values. We then calculated the upper 95% quantile for each rounded average baseline sensitivity in each eye, using only the baseline data.

Point-wise improvement was tested by comparing the proportion of locations exceeding the top 95% limits of repeatability expected from their average baseline sensitivity between treated eyes and those on placebo. The difference was tested with a logistic regression: each of the four follow-up test results for each location was considered an independent random trial, which could result in a sensitivity value above (success) or below (failure) the 95% baseline threshold. The multi-trial logistic regression coefficients, standard errors and *p*-values were estimated using generalized estimating equations (GEE), to account for correlations among locations from the same eye. Note that the sequential follow-up test results for the same location are already grouped in the multi-trial logistic regression framework (number of successes/4 trials per location).

Pearson’s correlation coefficients were calculated to evaluate linear correlations between the differences (3 months minus baseline) of PERG As with VEP IT ones. A *p*-value less than 0.01 was considered statistically significant.

SPSS (version 25), MedCalc V.13.0.4.0 (MedCalc, Mariakerke, Belgium) and R 4.5.1 (R Foundation for Statistical Computing, Vienna, Austria) were used for statistical analysis.

## 5. Conclusions

Our results suggest that in OAG patients, the treatment for 3 months with Citicoline (dosage of 400 mg/day) plus Nicotinamide (dosage of 150 mg/day) oral solution induces an enhancement of RGC function (PERG improvement), likely due to the mechanism of action of both components. With the same treatment, better neural conduction along the visual pathways (VEP improvement), likely due to the neuromodulator effect of Citicoline, is also evident. This treatment, when performed for 3 months, does not induce significant changes in visual field defects or in RNFL-T.

However, these findings should be interpreted with caution, as the short-term electrophysiological improvements observed following treatment with the Citicoline plus Nicotinamide oral solution cannot be assumed to reflect a reduction in the risk of glaucoma onset or a slowing of disease progression. Studies with longer follow-up and more comprehensive data collection are needed to confirm these findings in a chronic, lifelong disease such as glaucoma. Likewise, our results cannot be considered as conclusive and could encourage further studies to clarify whether the Nicotinamide dosage used in this study induces an RGC functional enhancement; a study performed by administering exclusively Nicotinamide at the dosage of 15 mg/mL is desirable.

It is interesting that, contrary to what has been observed in other studies with Nicotinamide, thanks to optimal dosage of the two molecules administered in this combination in oral solution, we showed an improvement in RGC function and neural conduction in visual pathways, with a good tolerance of Nicotinamide (150 mg/day) was observed. To confirm these findings, a study performed with long period of follow-up (at least one year) is required.

## Figures and Tables

**Table 1:** Mean values and one standard deviation (SD) of pattern electroretinogram (PERG) P50-N95 amplitudes, visual evoked potentials (VEP) P100 implicit times and VEP N75-P100 amplitudes detected at baseline and after 3 months in OAG patients treated with the placebo (Placebo group, 10 eyes) or Citicoline plus Nicotinamide (Citicoline plus Nicotinamide group, 18 eyes). 60′ and 15′ refer to visual stimuli in which each check subtended 60 min and 15 min of the visual arc, respectively. ANOVA: Statistical evaluation by a one-way analysis of variance, Citicoline plus Nicotinamide group versus Placebo group at baseline; Citicoline plus Nicotinamide group and Placebo group at follow-up evaluation (3 months) compared to baseline.

**PERG P50-N95 Amplitude (microVolt)**							
				**ANOVA ^a^ vs. Placebo**		**ANOVA ^a^ vs. Baseline**	
				**f**	***p***	**f**	***p***
**Groups**	**Time**	**Mean**	**SD ^b^**				
Placebo	Baseline	1.925	0.785				
Placebo	3 months	1.759	0.552			(1, 19) = 0.301	0.591
Citicoline plus Nicotinamide	Baseline	1.573	0.732	(1, 27) = 1.41	0.246		
Citicoline plus Nicotinamide	3 months	2.534	1.136			(1, 35) = 9.099	0.005
**60′ ^c^ VEP P100 Implicit Time (msec ^d^)**							
				**ANOVA ^a^ vs. Placebo**		**ANOVA ^a^ vs. Baseline**	
				**f**	***p***	**f**	***p***
**Groups**	**Time**	**Mean**	**SD ^b^**				
Placebo	Baseline	125.450	9.636				
Placebo	3 months	126.853	8.302			(1, 19) = 0.119	0.731
Citicoline plus Nicotinamide	Baseline	129.634	8.446	(1, 27) = 1.43	0.243		
Citicoline plus Nicotinamide	3 months	121.743	11.817			(1, 35) = 5.312	0.027
**60′ ^c^ VEP N75-P100 Amplitude (microVolt)**							
				**ANOVA ^a^ vs. Placebo**		**ANOVA ^a^ vs. Baseline**	
				**f**	***p***	**f**	***p***
**Groups**	**Time**	**Mean**	**SD ^b^**				
Placebo	Baseline	4.876	2.103				
Placebo	3 months	4.156	1.945			(1, 19) = 0.629	0.437
Citicoline plus Nicotinamide	Baseline	5.564	2.576	(1, 27) = 0.520	0.478		
Citicoline plus Nicotinamide	3 months	8.166	4.385			(1, 35) = 4.709	0.037
**15′ ^e^ VEP P100 Implicit Time (msec ^d^)**							
				**ANOVA ^a^ vs. Placebo**		**ANOVA ^a^ vs. Baseline**	
				**f**	***p***	**f**	***p***
**Groups**	**Time**	**Mean**	**SD ^b^**				
Placebo	Baseline	129.897	12.187				
Placebo	3 months	134.447	10.699			(1, 19) = 0.789	0.387
Citicoline plus Nicotinamide	Baseline	132.096	11.164	(1, 27) = 2.239	0.147		
Citicoline plus Nicotinamide	3 months	123.052	9.784			(1, 25) = 6.679	0.014
**15′ ^e^ VEP N75-P100 Amplitude (microVolt)**							
				**ANOVA ^a^ vs. Placebo**		**ANOVA ^a^ vs. Baseline**	
				**f**	***p***	**f**	***p***
**Groups**	**Time**	**Mean**	**SD ^b^**				
Placebo	Baseline	3.770	2.024				
Placebo	3 months	3.076	1.495			(1, 19) = 0.758	0.395
Citicoline plus Nicotinamide	Baseline	3.648	2.340	(1, 27) = 0.019	0.891		
Citicoline plus Nicotinamide	3 months	5.354	2.534			(1, 35) = 4.399	0.043

^a^ ANOVA = One-way analysis of variance; ^b^ SD = one standard deviation. ^c^ 60′ = visual stimuli in which each check subtended 60 min of the visual arc; ^d^ msec = milliseconds; ^e^ 15′ = visual stimuli in which each check subtended 15 min of the visual arc.

**Table 2:** Changes (difference 3 months minus baseline) in electrophysiological parameters (PERG P50-N95 amplitudes, VEP P100 implicit times and VEP N75-P100 amplitudes) after 3 months of treatment with respect to the baseline condition observed in OAG patients treated with the placebo (Placebo group) or Citicoline plus Nicotinamide (Citicoline plus Nicotinamide group).

Difference 3 Months Minus Baseline												
	Placebo Group (10 Eyes)						Citicoline Plus Nicotinamide Group (18 Eyes)					
	Unmodified		Improvement		Worsening		Unmodified		Improvement		Worsening	
	N	%	N	%	N	%	N	%	N	%	N	%
PERG P50-N95 A	7	70.00	1	10.00	2	20.00	3	16.67	15	83.33	0	0.00
60′ VEP P100 IT	7	70.00	0	0.00	3	30.00	7	38.89	11	61.11	0	0.00
60′ VEP N75-P100 A	7	70.00	0	0.00	3	30.00	6	33.33	12	66.67	0	0.00
15′ VEP P100 IT	8	80.00	0	0.00	2	20.00	7	38.89	10	55.56	1	5.56
15′ VEP N75-P100 A	8	80.00	0	0.00	2	20.00	7	38.89	11	61.11	0	0.00

Unmodified = within the 95% confidence test–retest limit; improvement = values with increase in amplitudes (A) or shortening in implicit times (IT) that exceeded the 95% confidence test–retest limit; worsening = values with reduced amplitudes (A) or increased implicit times (IT) that exceeded the 95% confidence test–retest limit; N = number of eyes; A = amplitude; 60′ = visual stimuli in which each check subtended 60 min of the visual arc; IT = implicit time; 15′ = visual stimuli in which each check subtended 15 min of the visual arc.

**Table 3:** Mean values and one standard deviation (SD) of the individual differences (3 months minus baseline) of pattern electroretinogram (PERG) P50-N95 amplitudes (PERG A), visual evoked potentials (VEP P100) implicit times (VEP IT) and VEP N75-P100 amplitudes (VEP A), Humphrey Field Analyzer (HFA) mean deviation, and retinal nerve fiber layer (RNFL) thickness detected in OAG patients treated with the placebo (Placebo group) or Citicoline plus Nicotinamide (Citicoline plus Nicotinamide group). 60′ and 15′ refer to visual stimuli in which each check subtended 60 min and 15 min of the visual arc, respectively. ANOVA: Statistical evaluation by a one-way analysis of variance, Citicoline plus Nicotinamide group versus Placebo group.

	Placebo Group		Citicoline Plus Nicotinamide Group		ANOVA ^a^ vs. Placebo	
	Mean	SD ^b^	Mean	SD ^b^	f	*p*
PERG A ^c^ (log ^d^ microVolt)	−0.022	0.088	0.218	0.158	19.489	<0.001
60′ ^e^ VEP IT ^f^ (log ^d^ msec ^g^)	0.005	0.049	−0.028	0.023	5.949	=0.022
60′ ^e^ VEP A ^c^ (log ^d^ microVolt)	−0.063	0.164	0.158	0.244	6.509	0.017
15′ ^h^ VEP IT ^f^ (log ^d^ msec ^g^)	0.015	0.020	−0.031	0.038	12.56	0.002
15′ ^h^ VEP A ^c^ (log ^d^ microVolt)	−0.084	0.159	0.191	0.179	16.37	<0.001

^a^ ANOVA = one-way analysis of variance; ^b^ SD = one standard deviation; ^c^ A = amplitude; ^d^ log = logarithm; ^e^ 60′ = visual stimuli in which each check subtended 60 min of the visual arc; ^f^ IT = implicit time; ^g^ msec = milliseconds; ^h^ 15′ = visual stimuli in which each check subtended 15 min of the visual arc.

## Data Availability

The raw data supporting the conclusions of this article will be made available by the authors on request.

## Supplementary Materials

The following supporting information can be downloaded at: https://www.mdpi.com/article/10.3390/ph19081179/s1, Figure S1: CONSORT flow diagram. CONSORT diagram reporting details concerning screening, enrolment, randomization and data analysis; Table S1: Baseline characteristics. Baseline characteristics and glaucoma medications of the study groups.

## Abbreviations and Acronyms

A	amplitude
ANOVA	one-way analysis of variance
BCVA	best corrected visual acuity
DB	decibel
DTI	diffusion tensor imaging
HFA	Humphrey Field Analyzer
IOP	intraocular pressure
ISCEV	International Society for Clinical Electrophysiology of Vision
IT	implicit time
LGN	lateral geniculate nucleus
logMAR	logarithm of the minimum angle of resolution
MD	mean deviation
MRI	magnetic resonance imaging
N	number of eyes of each group
NAD	Nicotinamide adenine dinucleotide
OAG	open angle glaucoma
OCT	optical coherence tomography
ONH	optic nerve head
PERG	pattern electroretinogram
PhNR	photopic negative response
RCT	retinocortical time
RGCs	retinal ganglion cells
RNFL	retinal nerve fiber layer
SD	one standard deviation of the mean
SD-OCT	spectral domain-optical coherence tomography
SNR	signal-to-noise ratio
TD	total deviation
VEP	visual evoked potentials
VF	visual field
