# Supplementary material for: Effects of Treatment with Citicoline Plus Nicotinamide Oral Solution on Retinal Ganglion Cells and Visual Pathways in Glaucomatous Impairment
Source: Pharmaceuticals (Basel). 2026 Jul 28;19(8):1179. doi: 10.3390/ph19081179 (PMC13516458; doi:10.3390/ph19081179)
Supplement: Supplementary file 1 [file pharmaceuticals-19-01179-s001.zip › Supplementary figure S1.pdf]

Supplementary figure S1

CONSORT Flow Diagram

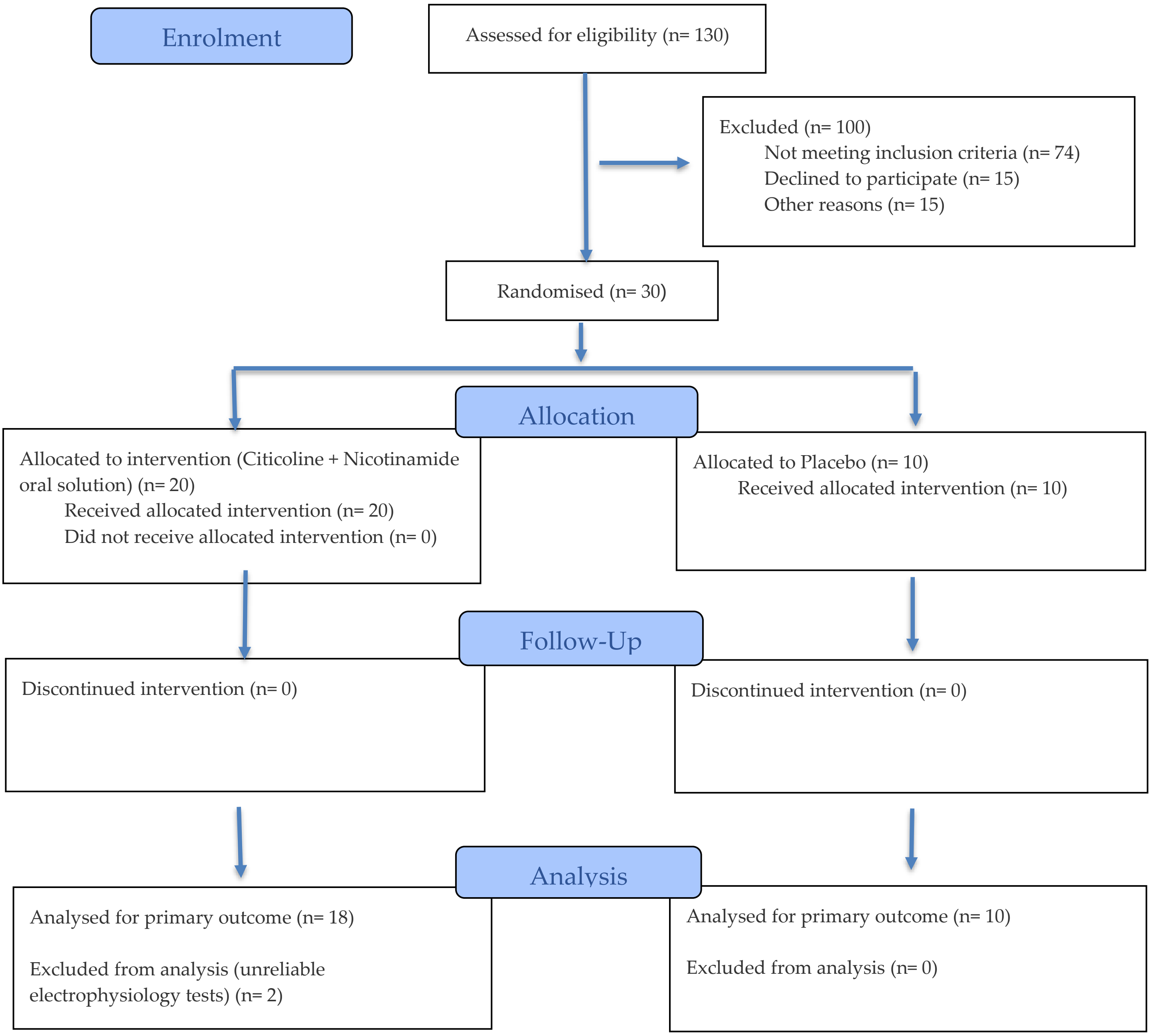

Citation: Hopewell S, Chan AW, Collins GS, Hróbjartsson A, Moher D, Schulz KF, et al. CONSORT 2025 Statement: updated guideline for reporting randomised trials. BMJ. 2025; 388:e081123. https://dx.doi.org/10.1136/bmj-2024-081123

© 2025 Hopewell et al. This is an Open Access article distributed under the terms of the Creative Commons Attribution License (https://creativecommons.org/licenses/by/4.0/), which permits unrestricted use, distribution, and reproduction in any medium, provided the original work is properly cited.
