# Supplementary material for: Effects of Treatment with Citicoline Plus Nicotinamide Oral Solution on Retinal Ganglion Cells and Visual Pathways in Glaucomatous Impairment
Source: Pharmaceuticals (Basel). 2026 Jul 28;19(8):1179. doi: 10.3390/ph19081179 (PMC13516458; doi:10.3390/ph19081179)
Supplement: Supplementary file 1 [file pharmaceuticals-19-01179-s001.zip › Supplementary table S1.pdf]

	Citiceline plus Nicotinamide group (20 eyes)	Placebo Group (10 eyes)	P value
IOP (mmHg +/- SD)	13.5 ± 1.8	13.0 ± 2.1	0.754
CCT (um +/- SD)	536 ± 29.4	523 ± 31.7	0.738
Number of drops (mean n ± SD)	2.0 ± 0.8	1.9 ± 0.7	0.717
Prostaglandin analogue (n (%))	19 (95%)	9 (90%)	1.00
β-blocker (n (%))	13 (65%)	6 (60%)	1.00
Topical CAI (n (%))	8 (40%)	4 (30%)	1.00
α2- agonist (n (%))	0 (0%)	0 (0%)	-
Previous laser treatment (n (%))	0 (0%)	0 (0%)	-
Previous glaucoma surgery (n (%))	0 (0%)	0 (0%)	-

Supplementary Table S1

Baseline characteristics of the two groups. Categorical variables were compared using Fisher's exact test, whereas continuous variables were compared using the independent-samples t-test or the Mann–Whitney U test, as appropriate.
